# The basal free fatty acid concentration in human saliva is related to salivary lipolytic activity

**DOI:** 10.1038/s41598-017-06418-2

**Published:** 2017-07-20

**Authors:** Eric Neyraud, Stéphanie Cabaret, Hélène Brignot, Claire Chabanet, Hélène Labouré, Elisabeth Guichard, Olivier Berdeaux

**Affiliations:** Centre des Sciences du Goût et de l’Alimentation, AgroSup Dijon, CNRS, INRA, Univ. Bourgogne Franche-Comté, Dijon, F-21000 France

## Abstract

Fat perception during eating is a complex sensation that involves various sensory modalities, such as texture, aroma and taste. Taste is supported by the discovery of fatty acid receptors in the tongue papillae. Dietary fat is mainly composed of esterified fatty acids, whereas only free fatty acids can bind to taste receptors. Some authors have mentioned the necessity and efficiency of salivary lipolytic activity to hydrolyse the esterified fatty acids present in foods and enable fat perception. Our hypothesis is that salivary lipolytic activity is also involved in regulating the basal level of salivary fatty acids in humans. To test this hypothesis, total fatty acid (TFA) and free fatty acid (FFA) concentrations and selected salivary characteristics (such as lipolytic activity) were analysed in the resting saliva of 54 subjects. The results show differences in the TFA and FFA profiles, with TFA and FFA concentrations of 8.99 and 3.56 µg/mL of saliva, respectively. Interestingly, lipolytic activity had a significant positive correlation with FFA concentration (0.51, p < 0.01). This result highlights a possible physiological role of salivary lipolytic activity in the regulation of the basal FFA concentration. This regulation could be involved in fat taste sensitivity.

## Introduction

Reducing fat consumption has become a key challenge for modern societies since the high consumption of fatty products has been associated with obesity and its associated pathologies, such as cardiovascular diseases, cancer, diabetes^1, 2^. However, the attraction of the consumer to fatty products is not well understood, and a strong variability in liking fat is observed in the population. This variability is partly explained by socio-economic, demographic, lifestyle-related, physiological and health-related characteristics^3–5^; however, it is also related to the sensory properties of fat^6^. Fat perception is a complex phenomenon involving various sensory modalities, such as the perception of texture, aroma and taste^7–9^. The taste perception of fat was suggested a few years ago with the discovery of long chain fatty acid receptors in the taste buds of the taste papillae in animal models; delayed rectifying K^+^ channels^10^, receptors belonging to the GPCR family, such as GPCR 40 and GPCR 120, and the CD36 receptor were found. The two latter types of receptors were also identified in human taste buds^11, 12^.

However, the taste perception of fatty acids is subject to controversy since dietary fat is mainly composed of triglycerides although it contains also free fatty acids. Therefore, it was postulated that the hydrolysis of triglycerides in the mouth is necessary to release free, perceivable fatty acids. The presence of a lipolytic activity in certain foods and in saliva makes this mechanism theoretically possible. Some studies have shown that, in resting saliva, there are positive relationships i) between lipolytic activity and fat perception^13^ and ii) between lipolytic activity and a preference for high-fat foods and dietary fat^14^. An interventional study has also shown that the inhibition of this lipolytic activity leads to a decrease in sensitivity to oleic acid taste^15^. However, for other authors, there was only weak evidence that lingual lipase is a determinant of oral fat detection^16^.

Considering that the debate of the role of salivary lipolytic activity in fat taste perception is still on-going, we propose a new hypothesis concerning the contribution of lipolytic activity in fat taste perception as linked to sensory adaptation. Indeed, the sensory adaptation of the taste receptors to salivary compounds would contribute to inter-individual variability in the sensitivity to different tastes, as was already described for the salty taste of sodium ions. Sodium taste receptors are indeed adapted to the concentration of sodium present in the saliva^17^. To be perceived, the sodium concentration in an exogenous sensory stimulus needs to be higher than its concentration in the saliva. We formed the hypothesis that the same mechanism could be involved in fat taste. Indeed, it is well known that human saliva contains various fatty acids in their esterified or non-esterified forms^18–23^. Adaptation to the free fatty acids present in saliva could be responsible, at least in part, to the sensitivity to exogenous fatty acids. Our hypothesis is that the salivary lipolytic activity can modulate the salivary free fatty acid concentration and thus impact perception. This paper constitutes a first step to test this hypothesis by studying the modulation of the basal concentration in fatty acids by the lipolytic activity in saliva.

To our knowledge, no publications to date have reported the concentrations of various fatty acids in their total forms (TFA): non-esterified + esterified (e.g. tri-, di- and mono-glycerides; phospholipids) and free forms (FFA): non-esterified. This lack of publications is probably due, in part, to the very low fatty acid concentrations in saliva. The first aim of the present work was to optimize a method for the extraction and quantification of free fatty acids (FFA) and total fatty acids (TFA) in saliva by GC-MS and GC-FID, respectively. The second aim was to study the relationships between FFA, TFA and other salivary characteristics that may affect the fatty acid profiles in saliva, particularly lipolytic activity.

## Materials and Methods

### Subject selection and ethics statement

Fifty-four subjects (28 women and 26 men) aged 24–74 years (average 51 years) participated in this study. The study was conducted according to the Declaration of Helsinki and the protocol was approved by the French ethical committee (Comité de Protection des Personnes Sud-Est I, n° 2013-A01378-37) and by the Agence Nationale de Sécurité du Médicament et des Produits de Santé (ANSM, n° 140061B-21). The subjects signed an informed consent form before participation and received compensation for their participation.

### Saliva sampling

Resting saliva was collected on 2 different days for each subject at 6 pm. The subjects were instructed to take a comfortable position, sitting and slightly leaning forward. The subjects had the instruction to let saliva accumulate naturally in their mouth without any stimulation or movement (tongue, cheeks) and to drool into a preweighed screw-cap cup whenever they felt like it. They were also instructed to keep focussing on the sampling and to avoid swallowing. This sampling was performed over a period of 10 min. This duration was necessary to obtain enough material for the fatty acid analyses due to the low amount of fatty acids in saliva. The subjects were asked not to eat, drink or smoke for at least one hour before the collection of saliva samples. The cups were weighed, and the salivary flow rates were expressed in mL/min, assuming that one g of saliva corresponds to one mL. Immediately after collection, the saliva samples were stored at −80 °C. Before biochemical analyses, for each subject, the 2 samples were thawed, pooled together, and centrifuged for 20 min at 14,000 g to remove bacteria and cellular debris.

### Saliva biochemical analyses

The *protein concentration* was measured using a Quick Start Bradford protein assay (Bio-Rad, France) with bovine serum albumin as the calibration standard.

The *total antioxidant capacity* was measured using an ORAC Assay kit (CellBiolabs, San Diego, CA). This assay measures the loss of fluorescence over time due to peroxyl-radical formation induced by the breakdown of 2,2′-azobis-2-methyl-propanimidamide, dihydrochloride (AAPH). This peroxyl radical oxidizes fluorescein, leading to a loss of fluorescence. Trolox [6-hydroxy-2,5,7,8-tetramethylchroman-2-carboxylic acid], a vitamin E analogue, served as a standard to inhibit fluorescein fluorescence decay in a dose-dependent manner. The intensity of fluorescence was measured (excitation filter: 485 nm, emission filter: 538 nm) with a microtiter plate fluorometer (Victor 3-V, Perkin Elmer, Waltham, MA, USA). The total antioxidant capacity of the saliva was expressed as the Trolox equivalent.

The *lipolytic activity* measurement was adapted from the method described in Neyraud *et al*.^13^. A 20 mM substrate solution was prepared from 4-methylumbelliferyl-7-oleate (Sigma–Aldrich, France) in ethanol and was diluted to 1 mM with a buffer containing 50 mM Tris–HCl, pH 7.5, 4 mM CaCl_2_, 2 mM EDTA, 0.2% (w/v) NaTDC (sodium taurodeoxycholate), 1 mM PMSF (phenylmethylsulphonyl fluoride), 1 mM DTT and 0.02% (w/v) sodium azide. The hydrolysis reaction was initiated by adding 37.5 μL of saliva to 150 μL of the 1 mM substrate solution and 1.5 μL ethanol. An inhibition reaction was also performed on each sample by adding 1.5 μL of a 56 mM ethanolic solution of THL (tetrahydrolipstatin) instead of ethanol. The fluorescence intensity was recorded by following the kinetics of the reaction at 37 °C (excitation filter: 355 nm; emission filter: 460 nm) using a microplate fluorometer (Victor 3-V, PerkinElmer, France). The lipolytic activity was calculated from the difference in the fluorescence slopes of each sample with and without THL and was expressed in reference to a standard curve of umbelliferone. At each set of measurements, a control of the linearity and proportionality of the reaction was performed using commercial lipase (Aspergillus niger Lipase, Fluka, France).

The *lipocalin concentration* (LCN-1) was determined using a Human LCN-1 ELISA Kit (CLOUD-CLONE CORP., TX, USA), as described in Mounayar *et al*.^24^.

### Saliva fatty acid analyses

#### Chemical products

HPLC-grade N-hexane, chloroform and methanol were purchased from Fisher Scientific (Illkirch, France). Pentafluorobenzyl bromide (PFB-Br), N,N diisopropylethylamine (DIPEA), boron trifluoride in 14% methanol (BF3-MeOH) and Supelco® 37 Component Fatty Acid Methyl Esters (FAME) Mix were obtained from Sigma Aldrich (Saint Quentin Fallavier, France). Standards of FFA were purchased from Matreya Biovalley (Conches, France).

#### Lipid extraction

The same extraction protocol was applied for the analysis of TFA and FFA. The saliva used for the extraction varied from 0.8 to 3 mL for TFA and from 0.8 to 1 mL for FFA, depending on the sample available. The saliva samples were lyophilized in Pyrex round bottom tubes after freezing at −80 °C for 8 h using a dry freezing system^25^. A mixture containing 0.5 mL of a water solution of 0.9% sodium chloride and 2.5 mL of a chloroform/methanol (2:1, v/v) solution was added to each lyophilized sample to extract total lipids (TL) according to Folch *et al*.^26^. Then, an internal standard (IS) containing 17:0 (2 µg/mL), 19:0 (0.5 µg/mL) and 23:0 (0.25 µg/mL) was added to the mixture. The mixture was homogenized for 5 min and centrifuged at 16,209 g for 3 min. The lower phase (containing TL) was recovered in a 10 mL tube. The aqueous phase was again extracted with 1.7 mL of chloroform. The two organic phases containing TL were combined, and the solvent was evaporated under a stream of nitrogen.

#### Analysis of TFA derived by gas chromatography – flame ionization detection (GC-FID)

Prior to GC-FID, TFA were transmethylated using BF3-MeOH according to Morrison and Smith^27^. FAME were dissolved in 100 µL of hexane and transferred to a vial with insert. GC-FID analyses were performed using a Hewlett Packard model 5890 gas chromatograph (Palo Alto, CA, USA) equipped with a split/splitless injector and an FID. The injector and the detector were maintained at 250 °C. FAME were analysed using a CPSIL-88 capillary column (100 m × 0.25 mm id film, thickness 0.20 µm; Varian, Les Ulis, France) under the following temperature program: 60 °C isothermal for 5 min, increased to 165 °C at 15 °C/min, isothermal for 1 min at this temperature, increased to 225 °C at 2 °C/min and held isothermal for 17 min at 225 °C^28^. The inlet pressure of the carrier gas (H2) was 200 kPa (velocity: 37.5 cm/s at 60 °C). FAME were detected and identified by comparison with commercial and synthetic standards (Supelco® 37 Component FAME Mix). For quantification, the ratio of the peak area of the FAME species to the peak area of the internal standards was used. The data were processed using EZChrom Elite software (Agilent Technologies, Massy, France). The results were expressed in micrograms of FA per 1 mL of saliva (µg/mL).

#### Analysis of FFA pentafluorobenzyl ester derivatives (PFB esters) by gas chromatography-mass spectrometry (GC-MS)

**Figure 1:**
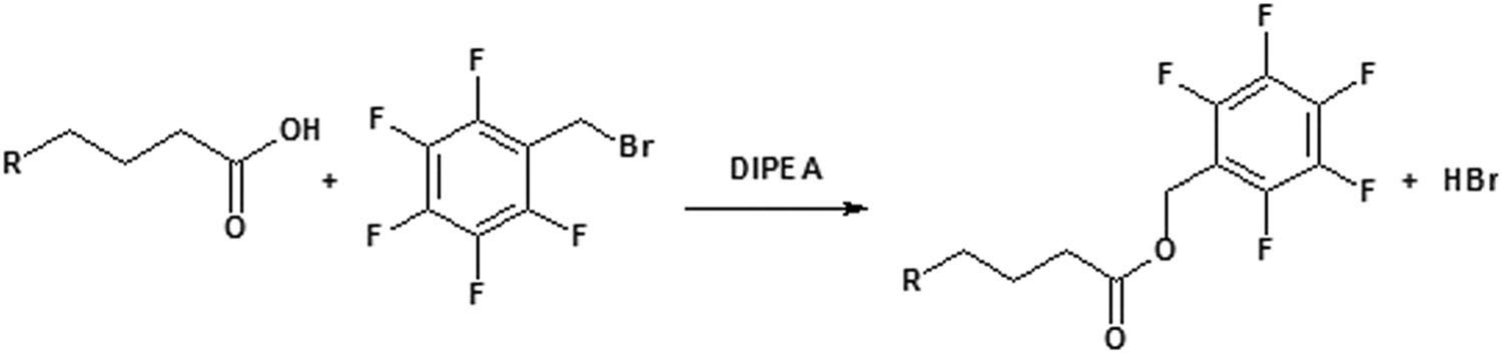
Reactions for the conversion of fatty acids into their pentafluorobenzyl esters using pentafluorobenzyl bromide (PFBBr).

FFA were converted to PFB esters for the GC-MS analysis. TL were treated with 100 μL of a solution of DIPEA in acetonitrile (1:50, v/v) and 100 μL of PFB-Br in acetonitrile (1:50, v/v) (Fig. 1). The mixture was vortexed for 1–2 min and left at room temperature for 30 min. The solvents and the reagents were removed under a stream of nitrogen. The PFB esters were dissolved in 100 µL of hexane and transferred to a vial with insert. The GC-MS analyses of PFB esters were carried out using a Shimadzu GC2010 gas chromatograph equipped with a split injector coupled to a Shimadzu GCMS-QP2010 mass spectrometer (Shimadzu) operating in the negative chemical ionization (NCI) mode with methane as reactive gas^29^. The electron energy was 70 eV, the source temperature was 230 °C, and the transfer line was 300 °C. The carboxylate anion [M-181]- of each detected PFB ester (Table 1) was used for quantification in the single ion monitoring mode (SIM)^30, 31^. Chromatography was carried out using an HP-5 MS fused silica capillary column (30 m × 0.25 mm internal diameter, 0.25 mm film thickness, Agilent Technologies) under the following temperature program: 50 °C isothermal for 0.5 min, increased to 140 °C at 5 °C/min, increased to 300 °C at 5 °C/min and held isothermal for 5 min at 300 °C. The flow rate of the carrier gas (H2) was 1.09 mL/min, and the temperature of the injector in the splitless mode was 250 °C. For quantification, the ratio of the peak area of each PFB ester species to the peak area of the internal standards was used. The results were expressed in micrograms of FA per mL of saliva.

**Table 1:** Chromatogram segments containing 4 to 7 carboxylate Ions [RCOO-] used in selected ion monitoring (SIM) for quantification of listed fatty acids.

Segment	Time start/end (min)	RCOO^−^ [M-181]^−^	Fatty acids	Fatty acids (IUPAC)
1	13.00	171,1	10:0	decanoic
		185,2	11:0	hendecanoic
		199,1	12:0	dodecanoic
		213,2	13:0	tridecanoic
		225,2	14:1	tetradecenoic (cis 9)
		227,1	14:0	tetradecanoic
	24.00	241,2	15:0	pentadecanoic
2	24.01	253,2	16:1	hexadecenoic (cis 7; cis 11)
		255,2	16:0	hexadecanoic
		267,2	17:1	heptadecenoic acid (cis 10)
	**27.00**	**269.2**	**17:0**	**heptadecanoic (SI)**
3	27.01	279.2	18:2	octadecadienoic (all cis 9,12)
		281.2	18:1	octadecenoic (cis 9; cis 11)
		277.2	18:3	octadecadienoic (all cis 6, 9, 12; all cis 9, 12, 15)
		275.2	18:4	octadecadienoic (all cis 6, 9, 12, 15)
		283.3	18:0	octadecanoic
	**29.70**	**297.3**	**19:0**	**nonadecanoic (SI)**
4	29.71	303.2	20:4	eicosatetraenoic (all cis 8, 11, 14, 17; all cis 5, 8, 11, 14)
		301.2	20:5	eicosapentaenoic (all cis 5,8, 11, 14, 17)
		305.2	20:3	eicosatrienoic (all cis 11, 14, 17)
		307.3	20:2	eicosadienoic (all cis 11, 14)
		309.3	20:1	eicosenoic (cis 11)
		305.2	20:3	eicosatrienoic (all cis 8, 11, 14; all cis 5, 8, 11)
	31.00	311.3	20:0	eicosanoic
5	31.01	325.3	21:0	heneicosanoic
		327.2	22:6	docosahexaenoic (all cis 4, 7, 10, 13, 16, 19)
		331.3	22:4	docosatetraenoic (all cis 7, 10, 13, 16)
		329.2	22:5	docosapentaenoic (all cis 7, 10, 13, 16, 19)
		337.3	22:1	docosenoic (cis 13)
	33.00	339.3	22:0	docosanoic
6	**33.01**	**353.3**	**23:0**	**tricosanoic (SI)**
		365.3	24:1	tetracosenoic (cis 15)
		367.4	24:0	tetracosanoic
	41.00	395.4	26:0	hexacosanoic

### Data analysis

The data were analysed using R Core Team (2015). R: A language and environment for statistical computing. R Foundation for Statistical Computing, Vienna, Austria (https://www.R-project.org) and the PLSS20.69R package(http://jf-durand-pls.com).

A logarithmic transformation was applied to all variables except flow to deal with skewed data. Regarding FFA concentrations, the quantity 0.005 (half of the smallest of the strictly positive values) was added to all measurements before transformation.

Pearson correlations between Lipolytic Activity and FFAs were calculated as well as 95% confidence intervals. A Bonferroni correction was finally applied to handle multiple testing.

The significant threshold (alpha) was set at 0.05.

## Results

**Figure 2:**
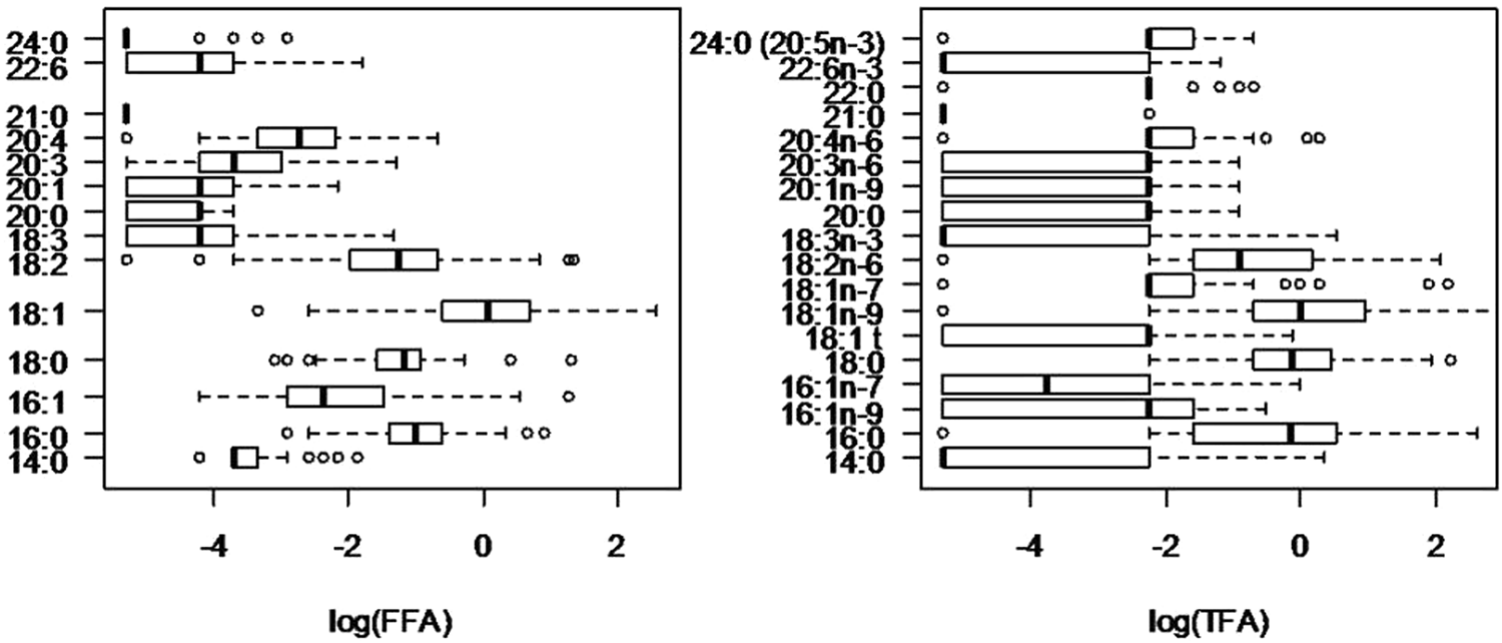
Box plot representations (n = 54) of Free fatty acids (FFA) on the left, and Total fatty acids (TFA) on the right. Logarithmic transformations were applied to all variables. Box outline represents the lower quartile and upper quartiles, the bold vertical line is the median value and outermost points correspond to observations that are far from the box (at a distance higher than 1.5 times the interquartile range).

Table 2 and Fig. 2 present the characteristics of the saliva from the 54 subjects. It is worth mentioning that the GC-MS method used for FFA profile determination does not allow classification of the unsaturated fatty acids based on the position of the unsaturated bond, contrarily to the GC-FID method used for TFA determination. Therefore, the values for each unsaturated FFA presented in Table 2 and Fig. 2 represent the sum of the various corresponding unsaturated fatty acids (e.g., free 18:1 corresponds to the sum of 18:1t, 18:1n-9, 18:1n-7). The most highly represented forms of both TFA and FFA are 18:1, followed by 18:0, 16:0 and 18:2. However, the sum of FFA corresponds to a concentration that is approximately 2 times lower than the sum of TFA, with respective average concentrations of 3.56 µg/mL (median; 2.32) and 8.99 µg/mL (median; 4.48).

**Table 2:** Characteristics of unstimulated saliva collected on 54 subjects.

**Salivary characteristics mesured**	**Median (Q1; Q3)**			
Unstimulated salivary flow (ml/min)	0.63 (0.41; 0.92)			
Protein concentration (mg/ml)	0.65 (0.45; 1.02)			
Lipolytic activity (mU/ml)	0.28 (0.16; 0.32)			
Antioxidant capacity (TROLOX µM/ml)	985 (600; 1783)			
Lipocalin concentration (ng/ml)	13.6 (9.9; 23.4)			
**Fatty acids**	**TFA**		**FFA**	
	**%**	**Concentration (µg/ml)**	**%**	**Concentration (µg/ml)**
	**Mean ± SD**	**Median (Q1; Q3)**	**Mean ± SD**	**Median (Q1; Q3)**
14:0	1.12 ± 1.87	0.00 (0.00; 0.11)	1.24 ± 1.00	0.02 (0.02; 0.03)
16:0	16.76 ± 9.14	0.85 (0.23; 1.68)	17.00 ± 8.75	0.37 (0.25; 0.53)
16:1 (16:1n-9; 16:1n-7)			6.41 ± 7.46	0.09 (0.05; 0.22)
16:1n-9	2.90 ± 3.65	0.10 (0.00; 0.20)		
16:1n-7	1.09 ± 0.76	0.05 (0.00; 0.10)		
18:0	19.79 ± 7.39	0.90 (0.50; 1.60)	13.20 ± 7.41	0.30 (0.20; 0.38)
18:1 (18:1 t; 18:1n-9; 18:1n-7)			44.80 ± 11.78	1.07 (0.58; 2.02)
18:1 t	1.47 ± 0.73	0.10 (0.00; 0.10)		
18:1n-9	25.99 ± 10.49	1.00 (0.50; 2.55)		
18:1n-7	3.40 ± 5.66	0.10 (0.10; 0.20)		
18:2n-6	10.58 ± 6.59	0.40 (0.20; 1.18)	11.68 ± 5.22	0.28 (0.14; 0.50)
18:3n-3	1.40 ± 1.50	0.00 (0.00; 0.10)	0.45 ± 0.42	0.01 (0.00; 0.02)
20:0	1.92 ± 1.34	0.10 (0.00; 0.10)	0.28 ± 0.31	0.01 (0.00; 0.01)
20:1n-9	2.12 ± 1.92	0.10 (0.00; 0.10)	0.49 ± 0.36	0.01 (0.00; 0.02)
20:3n-6	1.50 ± 1.60	0.10 (0.00; 0.10)	1.28 ± 0.82	0.02 (0.01; 0.04)
20:4n-6	2.95 ± 1.82	0.10 (0.10; 0.20)	2.37 ± 1.46	0.06 (0.03; 0.11)
21:0	0.31 ± 0.40	0.00 (0.00; 0.00)	0.00 ± 0.00	0.00 (0.00; 0.00)
22:0	2.56 ± 2.27	0.10 (0.10; 0.10)		
22:6n-3	0.95 ± 0.69	0.00 (0.00; 0.10)	0.51 ± 0.61	0.01 (0.00; 0.02)
24:0			0.29 ± 0.65	0.00 (0.00; 0.00)
24:0 (20:5n-3)	3.17 ± 2.44	0.10 (0.10; 0.20)		
Total	100	4.48 (2.6; 9.16)	100	2.32 (1.63; 4.00)

Free fatty acids 16:1 and 18:1 are presented as the sum of their isomers since GC-MS does not distinguish them. 24:0 and 20:5n-3 cannot be separated by GC-FID. Fatty acids composition for TFA and FFA is expressed in the mean ± SD relative percentage of each fatty acid among all the fatty acids detected and in the median (Q1, first quartile; Q3, third quartile) of the concentration of each fatty acid (µg/ml of saliva).

**Figure 3:**
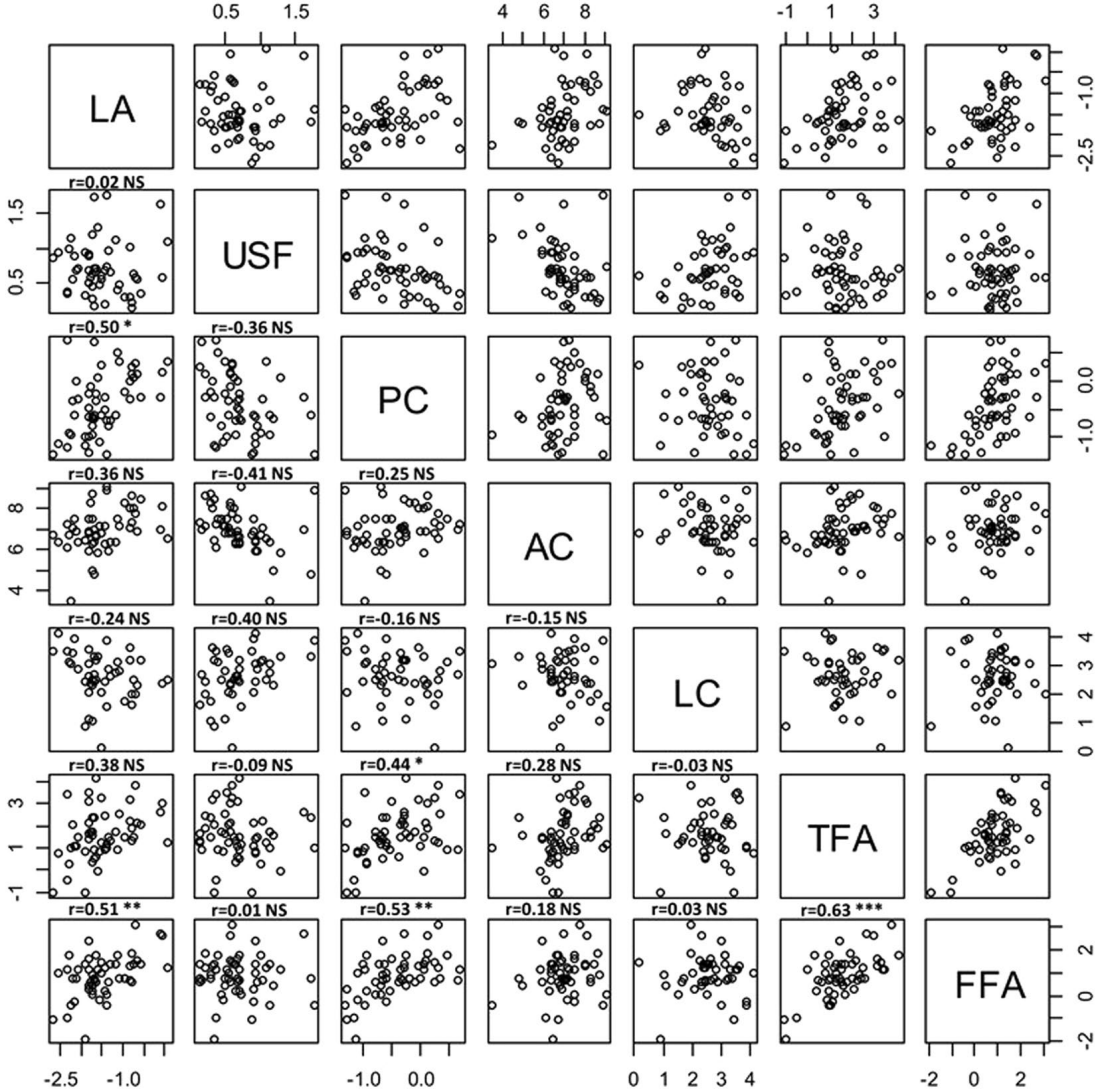
Scatterplot matrix of the different salivary variables (LA, Lipolytic Activity; USF, Unstimulated Salivary Flow; PC, Protein Concentration; AC, Antioxidant Capacity; LC, Lipocalin Concentration; TFA, Total Fatty Acids concentration; FFA, Free Fatty Acids concentration). Logarithmic transformations were applied to all variables except USF to deal with skewed data. Pearson coefficient is reported when adjusted p value (Bonferroni) is significant. NS: Non Significant; *p < 0.05; **p < 0.01; ***p < 0.001

The correlations between the different salivary characteristics are presented on the scatterplot matrix shown in Fig. 3. Protein concentration is positively correlated with lipolytic activity as well as TFA and FFA concentrations. TFA and FFA concentrations are positively correlated with one another, and interestingly, lipolytic activity is positively correlated with FFA concentration.

**Figure 4:**
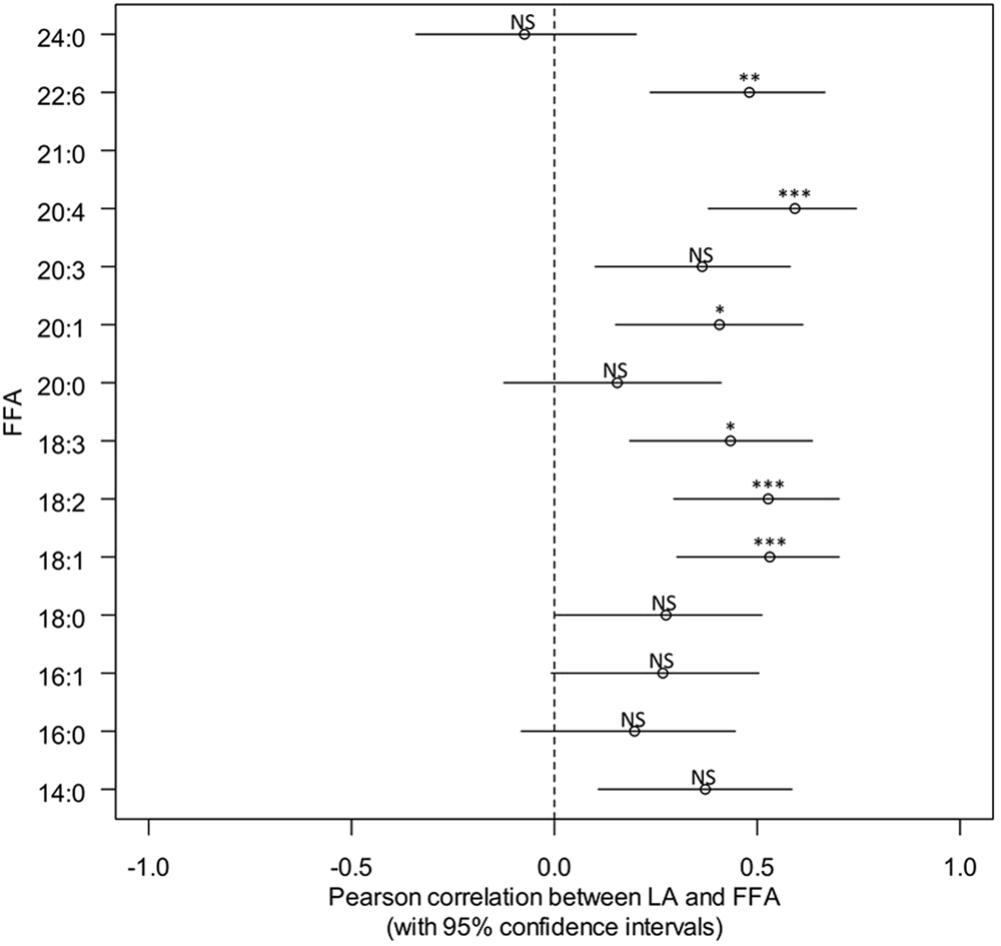
Pearson correlations between LA (Lipolytic Activity) and the different FFA (Free Fatty Acids). Logarithmic transformations were applied to all variables to deal with skewed data. Pearson coefficient is reported when adjusted p value (Bonferroni) is significant. NS: Non Significant; *p < 0.05; **p < 0.01; ***p < 0.001

The individual correlations between lipolytic activity and the different FFA concentrations are presented in Fig. 4 with 95% confidence intervals. Six fatty acids show a significant positive correlation with lipolytic activity after adjustment for multiple testing.

## Discussion

The aims of the present work were to determine the free (FFA) and total (TFA) fatty acid concentrations in human saliva and to study the relationships among FFA, TFA and other salivary characteristics that may affect the fatty acid profiles in saliva, particularly lipolytic activity. To achieve this aim, we first used an improved method for the extraction of fatty acids in saliva and their quantification (GC-FID for TFA and GC-MS for FFA). Our results on the percentage of TFA concur with those from previous studies^18, 21^, showing the highest abundance for 18:1. Other studies found that the most abundant fatty acids were either 16:0^20^ or 18:0^19^. However, there is agreement that the 4 most abundant fatty acids in saliva are 16:0; 18:0; 18:1 and 18:2. Reich *et al*.^21^ also determined the amount of TFA in saliva and found that concentrations vary from 1 to 10 µg/ml of saliva, which is in the range of our results. In the present work, we determined the concentration of FFA in saliva. We found a relatively elevated amount of FFA in saliva, representing approximately 50% of the TFA. Such a high proportion of FFA is in line with the hypothesis that the lipolytic activity of saliva could be responsible for the formation of FFA from the salivary endogenous esterified fatty acids. This hypothesis is supported by the significant positive correlation that we found between the lipolytic activity in saliva and FFA concentration (R = 0.51; p ≤ 0.01), whereas lipolytic activity was not significantly correlated with TFA concentration. Lipolytic activity in human saliva remains an enigma since the responsible enzyme has not yet been identified even if some potential lipase gene candidates were identified in an extensive work from Voigt *et al*.^32^. However, lipolytic activity has been often mentioned in human saliva during recent years by various groups^13, 15, 24, 33^. The physiological role of the lipolytic activity in saliva is not yet clear. The main hypothesis is about its potential role in our taste perception of fatty acids. Indeed, some authors formed the hypothesis that salivary lipolytic activity could allow the release of a sufficient amount of FFA from dietary fats, which are mainly composed of fatty acids in the esterified form^8^, in order to be perceived by the fatty acid taste receptors. This hypothesis is supported by the fact that sensitivity to the taste of triglycerides, which is actually the taste of free fatty acids released when triglycerides are hydrolysed, decreases when lipolytic activity is inhibited^15^. This result suggests that, in the absence of lipolytic activity, the amount of FFA released during food mastication is below the concentration required for the taste detection of FFA^34^. Even if this hypothesis is realistic, we propose a complementary hypothesis related to the modulation of basal FFA concentrations in saliva by salivary lipolytic activity. Indeed, sensory taste receptors could be adapted to the concentration of endogenous taste compounds in saliva. This phenomenon is well known for the taste of sodium^17^, since the detection threshold for sodium is higher than the sodium concentration in saliva. We believe that the same phenomenon occurs for fatty acids and that fatty acid taste sensitivity could be modulated by the FFA concentration in saliva. In the case of oleic acid (C18:1), the most studied fatty acid in fat taste studies, we found an average concentration in saliva of 1.73 µg/ml (approximately 6.1 µM). This concentration is below the average taste detection threshold reported in several studies (25.7 mM^35^, 19.9 mM^36^, and between 0.1 and 0.4 mM^12^) and this detection threshold varies importantly in literature (see Running *et al*. for review^37^), but Kulkarni and Mattes^34^ reported that some subjects have a detection threshold below this concentration (2 out of 15 subjects in their study). Therefore, it seems possible that the taste receptors for oleic acids can be adapted to the concentration of oleic acid in saliva, with a potential impact on its sensory detection.

We also measured other salivary characteristics, and the present study provided important information on their relationships with salivary fatty acid composition. These characteristics were antioxidant capacity, which could be involved in the oxidation of unsaturated fatty acids, total protein concentration and specific lipocalin (LCN-1) concentration. LCN-1 is also known in saliva as von Ebner’s gland protein (VEGP); LCN-1 can bind fatty acids and has been proposed to play the role of a fatty acid transporter in saliva^38^. However, this hypothesis has not been tested yet. In addition to lipolytic activity, we found that only 2 other salivary characteristics were significantly correlated with FFA: TFA and protein concentration. The relationships found between FFA and TFA can be explained by the fact that FFA represents half of the TFA. However, the correlation between fatty acids and protein concentration remains too complicated to explain since the protein composition of saliva is complex, with more than 4,000 proteins identified to date^39^; it is much too speculative to form any hypotheses on this relationship. Interestingly, we did not find significant correlations between antioxidant capacity and unsaturated fatty acids (results not shown). This result suggests that the contribution of the salivary antioxidant capacity towards the protection of unsaturated fatty acids against oxidation is negligible in resting saliva.

Altogether, this study shows for the first time that lipolytic activity is significantly correlated with the free fatty acid concentration in the saliva of human subjects, suggesting that lipolytic activity modulates the basal free fatty acid pattern in saliva. We propose the hypothesis that this modulation could have a physiological role, particularly in the taste perception of fatty acids, even if this role remains to be elucidated and this paper constitutes a first step to test this hypothesis.

## References

[1] Lampure A (2016). Associations between liking for fat, sweet or salt and obesity risk in French adults: a prospective cohort study. Int. J. Behav. Nutr. Phys. Act..

[2] Organization, W. H. Diet, Nutrition and the Prevention of Chronic Diseases. Report No. TRS 916, (Geneva, 2003). 12768890

[3] Lampure A (2014). Liking for fat is associated with sociodemographic, psychological, lifestyle and health characteristics. Br. J. Nutr..

[4] Mejean C, Macouillard P, Castetbon K, Kesse-Guyot E, Hercberg S (2011). Socio-economic, demographic, lifestyle and health characteristics associated with consumption of fatty-sweetened and fatty-salted foods in middle-aged French adults. Br. J. Nutr..

[5] Heinze JM, Preissl H, Fritsche A, Frank S (2015). Controversies in fat perception. Physiol. Behav..

[6] Liu DL, Archer N, Duesing K, Hannan G, Keast R (2016). Mechanism of fat taste perception: Association with diet and obesity. Prog. Lipid Res..

[7] Chale-Rush A, Burgess JR, Mattes RD (2007). Multiple routes of chemosensitivity to free fatty acids in humans. Am. J. Physiol.-Gastroint. Liver Physiol..

[8] Mattes RD (2009). Is There a Fatty Acid Taste?. Annu. Rev. Nutr..

[9] Martin C (2016). Sensory properties linked to fat content and tasting temperature in cottage cheese. Dairy Sci. Technol..

[10] Gilbertson TA (1998). Gustatory mechanisms for the detection of fat. Curr. Opin. Neurobiol..

[11] Simons PJ, Kummer JA, Luiken J, Boon L (2011). Apical CD36 immunolocalization in human and porcine taste buds from circumvallate and foliate papillae. Acta Histochem..

[12] Galindo MM (2012). G Protein-Coupled Receptors in Human Fat Taste Perception. Chemical Senses.

[13] Neyraud E, Palicki O, Schwartz C, Nicklaus S, Feron G (2012). Variability of human saliva composition: Possible relationships with fat perception and liking. Arch. Oral Biol..

[14] Mennella I, Fogliano V, Vitaglione P (2014). Salivary lipase and et-amylase activities are higher in overweight than in normal weight subjects: Influences on dietary behavior. Food Res. Int..

[15] Pepino MY, Love-Gregory L, Klein S, Abumrad NA (2012). The fatty acid translocase gene CD36 and lingual lipase influence oral sensitivity to fat in obese subjects. J. Lipid Res..

[16] Kulkarni BV, Mattes RD (2014). Lingual lipase activity in the orosensory detection of fat by humans. Am. J. Physiol.-Regul. Integr. Comp. Physiol..

[17] Bartoshuk LM (1978). Psychophysics of Taste. American Journal of Clinical Nutrition.

[18] Neyraud E, Tremblay-Franco M, Gregoire S, Berdeaux O, Canlet C (2013). Relationships between the metabolome and the fatty acid composition of human saliva; effects of stimulation. Metabolomics.

[19] Tomita Y, Miyake N, Yamanaka S (2008). Lipids in Human Parotid Saliva with Regard to Caries Experience. Journal of Oleo Science.

[20] Actis AB, Perovic NR, Defago D, Beccacece C, Eynard AR (2005). Fatty acid profile of human saliva: a possible indicator of dietary fat intake. Arch. Oral Biol..

[21] Reich M, Kummerer K, Al-Ahmad A, Hannig C (2013). Fatty Acid Profile of the Initial Oral Biofilm (Pellicle): an *In-Situ* Study. Lipids.

[22] Slomiany BL, Murty VLN, Aono M, Slomiany A, Mandel ID (1982). Lipid-composition of human-parotid and sub-mandibular saliva from caries-resistant and caries-susceptible adults. Arch. Oral Biol..

[23] Slomiany BL (1986). Lipid-composition and viscosity of parotid-saliva in Sjogren syndrome in man. Arch. Oral Biol..

[24] Mounayar R, Septier C, Chabanet C, Feron G, Neyraud E (2013). Oral Fat Sensitivity in Humans: Links to Saliva Composition Before and After Stimulation by Oleic Acid. Chemosensory Perception.

[25] Kulkarni, B. V., Wood, K. V. & Mattes, R. D. Quantitative and qualitative analyses of human salivary NEFA with gas-chromatography and mass spectrometry. *Front. Physiol*. **3**, 10.3389/fphys.2012.00328 (2012). 10.3389/fphys.2012.00328 PMC3429096 22934076

[26] Folch J, Lees M, Stanley GHS (1957). A simple method for the isolation and purification of total lipids from animal tissues. Journal of Biological Chemistry.

[27] Morrison WR, Smith LM (1964). Preparation of fatty acid methyl esters and dimethylacetals from lipids with boron fluoride-methanol. J. Lipid Res..

[28] Dionisi F, Golay PA, Fay LB (2002). Influence of milk fat presence on the determination of trans fatty acids in fats used for infant formulae. Anal. Chim. Acta.

[29] Pawlosky RJ, Sprecher HW, Salem N (1992). High sensitivity negative ion GC-MS method for detection of desaturated and chain-elongated products of deuterated linoleic and linolenic acids. J Lipid Res.

[30] Lee HB, Peart TE, Carron JM (1990). Gas-chromatographic and mass-spectrometric determination of some resin and fatty-acids in pulpmill effluents as their pentafluorobenzyl ester derivatives. Journal of Chromatography.

[31] Arnauld S (2009). Modulation of the hepatic fatty acid pool in peroxisomal 3-ketoacyl-CoA thiolase B-null mice exposed to the selective PPARalpha agonist Wy14,643. Biochimie.

[32] Voigt N (2014). The role of lipolysis in human orosensory fat perception. J. Lipid Res..

[33] Stewart JE (2010). Oral sensitivity to fatty acids, food consumption and BMI in human subjects. Br. J. Nutr..

[34] Kulkarni B, Mattes R (2013). Evidence for Presence of Nonesterified Fatty Acids as Potential Gustatory Signaling Molecules in Humans. Chemical Senses.

[35] Running CA, Mattes RD (2014). Different oral sensitivities to and sensations of short-, medium-, and long-chain fatty acids in humans. Am. J. Physiol.-Gastroint. Liver Physiol..

[36] Running CA, Mattes RD (2015). Humans are more sensitive to the taste of linoleic and alpha-linolenic than oleic acid. Am. J. Physiol.-Gastroint. Liver Physiol..

[37] Running CA, Mattes RD, Tucker RM (2013). Fat taste in humans: Sources of within- and between-subject variability. Prog. Lipid Res..

[38] Neyraud, E. In *Saliva: Secretion and Functions* Vol. 24 *Monographs in Oral Science* (eds A. J. M. Ligtenberg & E. C. I. Veerman) 61-70 (Karger, 2014).

[39] Denny P (2008). The proteomes of human parotid and submandibular/sublingual gland salivas collected as the ductal secretions. J. Proteome Res..

